# Distinct Roles of mTOR Targets S6K1 and S6K2 in Breast Cancer

**DOI:** 10.3390/ijms21041199

**Published:** 2020-02-11

**Authors:** Savitha Sridharan, Alakananda Basu

**Affiliations:** Department of Microbiology, Immunology and Genetics, University of North Texas Health Science Center, Fort Worth, TX 76107, USA; Savitha.Sridharan@live.unthsc.edu

**Keywords:** mTOR, S6K1, S6K2, *RPS6KB1*, *RPS6KB2*, breast cancer

## Abstract

The mechanistic target of rapamycin (mTOR) is a master regulator of protein translation, metabolism, cell growth and proliferation. It forms two complexes, mTOR complex 1 (mTORC1) and 2 (mTORC2). mTORC1 is frequently deregulated in many cancers, including breast cancer, and is an important target for cancer therapy. The immunosuppressant drug rapamycin and its analogs that inhibit mTOR are currently being evaluated for their potential as anti-cancer agents, albeit with limited efficacy. mTORC1 mediates its function via its downstream targets 40S ribosomal S6 kinases (S6K) and eukaryotic translation initiation factor 4E (eIF4E)-binding protein 1 (4E-BP1). There are two homologs of S6K: S6K1 and S6K2. Most of the earlier studies focused on S6K1 rather than S6K2. Because of their high degree of structural homology, it was generally believed that they behave similarly. Recent studies suggest that while they may share some functions, they may also exhibit distinct or even opposite functions. Both homologs have been implicated in breast cancer, although how they contribute to breast cancer may differ. The purpose of this review article is to compare and contrast the expression, structure, regulation and function of these two S6K homologs in breast cancer.

## 1. Introduction

Breast cancer is the second leading cause of cancer-related death among women in the United States. Multiple factors contribute to the poor survival and the severity of breast cancer. The mechanistic target of rapamycin (mTOR), also known as the mammalian target of rapamycin, is an important target for breast cancer therapy since it is frequently deregulated in breast cancers and plays a critical role in tumorigenesis [1,2]. mTOR forms two distinct complexes with either raptor (mTORC1) or rictor (mTORC2). mTORC1 acts downstream of the Akt signaling pathway, which is deregulated in approximately 60% of breast cancers and plays critical roles in breast cancer development, progression and resistance to chemotherapeutic drugs [2,3,4,5,6,7,8,9,10,11]. mTORC1 mediates its function via its downstream targets 40S ribosomal S6 kinase (S6K) and 4E-binding protein 1 (4E-BP1) [12].

Studies addressing the highly conserved inducible phosphorylation of ribosomal protein S6 in somatic cells led to the discovery of S6K1 (p70S6Kα) or p70S6 kinase [13,14]. It was originally identified as the serine/threonine kinase that mediated the mitogen-inducible phosphorylation of ribosomal protein S6 (rpS6). However, the observation that rpS6 phosphorylation was not affected in S6K1 knockout mice led to the identification of a close homolog of S6K1, S6K2 (p70S6Kβ), which was later shown to be the major kinase mediating rpS6 phosphorylation [15,16,17,18,19]. S6K1 and S6K2 are encoded by *RPS6KB1* on chromosome 17 and *RPS6KB2* on chromosome 11, respectively (Table 1). Both genes code for two isoforms each with the use of alternative translation start sites: p70 S6K (S6KαII) and p85 S6K (S6KαI) in the case of S6K1, and p54 S6K (S6KβII) and p56 S6K (S6KβI) for S6K2 [16,20]. The N-terminal extensions of the longer forms of both S6K1 and S6K2 harbor a functional nuclear localization signal (NLS), making them constitutively nuclear. However, the shorter isoforms represent the predominant forms for both homologs and will be referred to as S6K1 and S6K2 henceforth.

Most of the earlier studies focused on p70 S6K1 or S6K1 as the downstream target of mTORC1. It was believed that due to structural similarities, S6K1 and S6K2 share redundant functions. Recent studies, however, challenge this notion [21,22,23,24,25]. Both homologs have been implicated in breast cancer, although they may play distinct roles [26,27]. In this review article, we briefly describe differences in structural aspects, regulation and cellular functions of these two homologs prior to discussing their distinct roles in breast cancer.

## 2. Structure of S6Ks

**Figure 1:**
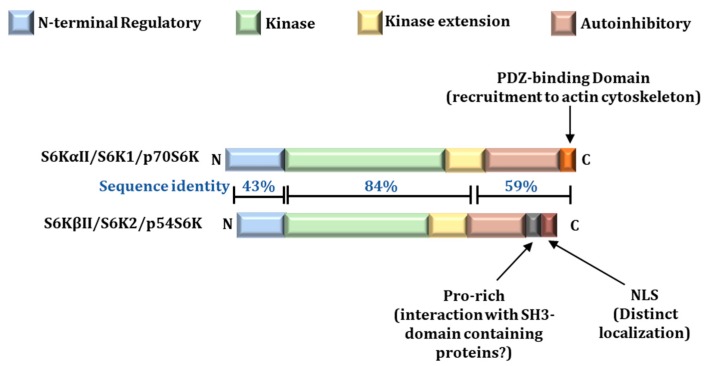
Modular structure of S6Ks. S6K1 and S6K2 share significant homology in their kinase domains. However, the shorter isoforms of both S6K1 and S6K2, which are the predominant forms, exhibit substantial divergence in the extreme N- and C-terminal regions.

Both S6K1 and S6K2 exhibit a modular structure consisting of an N-terminal regulatory region, the kinase domain, followed by the kinase extension domain and a C-terminal regulatory region harboring the autoinhibitory/pseudosubstrate domain (Figure 1). While they share over 80% homology in the amino acid sequence of their kinase domains, as well as a high degree of similarity in the adjacent kinase extension and pseudosubstrate or autoinhibitory domains with conserved sites critical for their activation [16,17], important differences exist in the extreme N- and C-terminal regions. S6K1 possesses a C-terminal PDZ-binding domain, which promotes association with the actin cytoskeleton [28], whereas S6K2 but not S6K1 harbors a functional nuclear localization signal (NLS) and a proline-rich domain, which may promote interaction with the SH3-domain containing proteins at its C-terminus [16] (Figure 1). It is believed that these key differences between S6K1 and S6K2 will result in differential localization and binding partners and hence distinct functions for the two proteins [21].

## 3. Regulation of S6Ks

### 3.1. Activation of S6Ks

**Figure 2:**
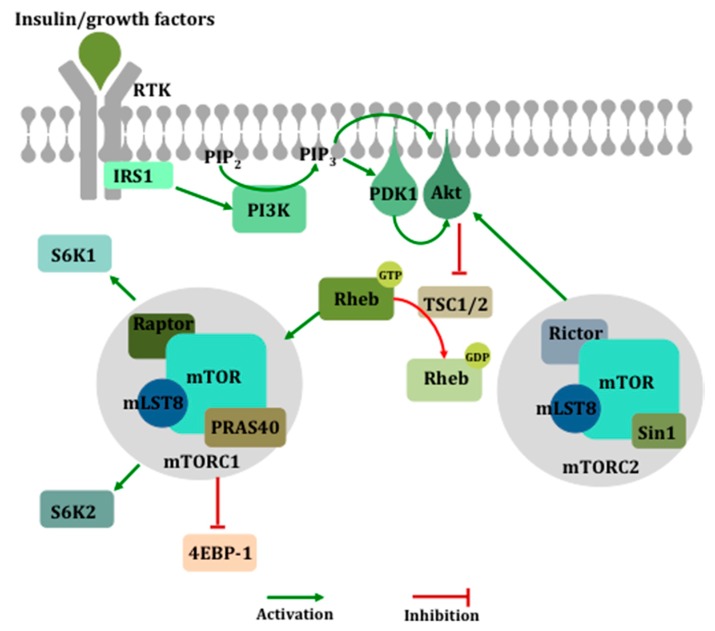
The activation of the mechanistic target of rapamycin (mTOR). Growth factor-mediated activation of the phosphatidylinositol 3-kinase (PI3K) pathway leads to the membrane recruitment and activation of phosphoinositide-dependent kinase 1 (PDK1) and Akt, which then phosphorylates and inactivates the tuberous sclerosis complex (TSC1/2), a negative regulator of ras homolog enriched in brain (Rheb), ultimately resulting in the activation of mTOR within complex 1. mTORC1 mediates its downstream effects primarily via the inhibition of eukaryotic translation initiation factor 4E (eIF4E)-binding protein 1 (4E-BP1) and the activation of S6 kinase (S6K).

Growth factor- and hormone-mediated activation of receptor tyrosine kinases promotes phosphatidylinositol 3-kinase (PI3K) activation, which then phosphorylates phosphatidylinositol-4,5-bisphosphate (PIP2) to produce phosphatidylinositol-3,4,5-trisphosphate (PIP3). This leads to the membrane recruitment and activation of pleckstrin homology domain-containing proteins such as phosphoinositide-dependent kinase 1 (PDK1). Activation of PDK1 leads to the phosphorylation and activation of several drivers of cell survival and proliferation such as Akt, which then promotes mTORC1 activation by negatively regulating the tuberous sclerosis complex (TSC), a tumor suppressor complex [29] mutated in hamartomas [30]. Inhibition of the TSC allows activation of the small GTPase ras homolog enriched in brain (RHEB) [31,32], and subsequent mTORC1 activation results in downstream signaling and cap-dependent translation by phosphorylating and inhibiting the eIF4E-binding protein (4E-BP) and activating S6K (Figure 2).

The sensitivity of S6K to the immunosuppressant drug rapamycin implied its regulation by mTOR [33,34,35]. Further studies suggested that the phospho-mimetic mutation of a conserved phosphorylation site in the kinase-extension domain (T389 in S6K1 and T388 in S6K2), known as the hydrophobic motif, by mTOR leads to resistance to rapamycin [36]. Raptor within the mTORC1 complex binds to the tor signaling motif (TOS), a conserved amino acid sequence found in S6K [37], and promotes its interaction with mTOR, which mediates the hydrophobic motif phosphorylation [38]. The activation of S6K is then achieved by PDK-1-mediated phosphorylation at a threonine residue in the activation loop (T229 in S6K1 and T228 in S6K2) within the kinase domain [39]. However, in order for mTORC1 and PDK1 to be able to access their target sites, a series of phosphorylation events at C-terminal serine residues first needs to occur so as to render the pseudosubstrate/autoinhibitory domain inactive and expose the activation loop, making it accessible for PDK1. The C-terminal autoinhibitory domain phosphorylations are believed to be carried out by members of the mitogen-activated protein kinase (MAPK) family [40]. Thus, the current model of S6K activation follows that the initial phosphorylation events in the C-terminal pseudosubstrate domain expose the kinase extension and kinase domains and promote mTORC1-mediated phosphorylation followed by the activating phosphorylation by PDK1 [21,23].

While S6K1 and S6K2 share the majority of conserved phosphorylation sites, they are believed to exhibit differences in their sensitivities to rapamycin and inputs from the MAPK signaling pathway. For example, in H510 lung cancer cells, which are characterized by highly active mitogen-activated protein kinase kinase (MEK) signaling, S6K2 was less responsive to rapamycin but highly sensitive to MEK inhibition [41]. The MAPK-mediated C-terminal phosphorylations appear to be more critical for the activation of S6K2 than that of S6K1 [41,42]. Furthermore, leucine deprivation affected S6K1 but not S6K2 or S6 phosphorylation, suggesting the differential regulation of the two kinases.

Growth factor-mediated activation of S6K1 is followed by its rapid dephosphorylation and desensitization via the serine/threonine protein phosphatase PP2A [43,44,45,46]. In addition to the phosphorylation and activation of S6K, mTOR leads to increases in levels of the serine/threonine protein phosphatase PHLPP (pleckstrin homology domain leucine-rich repeat protein phosphatase) [47], which aids in switching off S6K activity by dephosphorylating the hydrophobic motif [48]. Thus, functional mTOR not only promotes the activation of S6K but also ensures tight regulation of its activity via dephosphorylation.

### 3.2. Subcellular Localization, Tissue Distribution and Protein Turnover

The presence of a nuclear localization sequence at its C-terminus suggests S6K2 but not S6K1 is nuclear, where its activation can be regulated by a pool of nuclear mTOR [49]. Several phosphorylation events have been linked to the regulation of S6K localization. For example, S6K2 has been shown to shuttle between the nucleus and cytoplasm, which is believed to be regulated by growth factor-induced phosphorylation by protein kinase C (PKC) in its C-terminus close to its nuclear localization signal, thus inhibiting its nuclear translocation. This is believed to maintain a pool of active S6K2 in the cytoplasm [50]. Similarly, the phosphorylation of S6K1 by casein kinase 2 prevents its nuclear translocation [51], which was shown to occur upon growth factor stimulation despite the lack of a nuclear localization signal [51,52]. While these phosphorylation events do not directly correlate with the activity of S6Ks, they are believed to recruit them to specific cellular compartments or serve to bring them into the proximity of their targets, thus determining their functions. S6 kinases have also been shown to undergo tyrosine phosphorylation following their membrane recruitment via a platelet-derived growth factor receptor (PDGFR)-Src pathway [53]. However, the precise function of this phosphorylation event remains unclear.

While S6K1 and S6K2 mRNAs were ubiquitously expressed in all tissues tested, S6K2 mRNA and protein levels did not correlate with each other, prompting the authors to suggest the existence of post-transcriptional fine-tuning of tissue-specificity for S6K2 [54]. Recently, microRNA-mediated regulation of S6K2 has come into light in non-small cell lung cancers, where it was shown to be targeted by miR-193a-3p [55].

S6Ks are also regulated via protein degradation and stabilization. Both S6K1 and S6K2 have been reported to be ubiquitinated and degraded via the proteasome. While the Roc1 ubiquitin ligase was shown to ubiquitinate S6K1, the identity of the ubiquitin ligase mediating the proteasomal degradation of S6K2 remains unknown [56,57]. Furthermore, S6Ks have been shown to be acetylated by p300 in vitro and the inhibition of histone deacetylase in cells increased their levels, suggesting that S6K acetylation promotes their stabilization [58].

Thus, while the core activation mechanisms are conserved between S6K1 and S6K2, there exist important differences in their sensitivities to upstream signaling pathways, localization and modes of protein turnover.

## 4. Cellular Functions of S6Ks

Initial studies on the physiological role of S6K came from *Drosophila,* which possesses a single S6K (*dS6K)* gene [59]. The disruption of this gene decreases the probability of survival to adulthood with a marked decrease in body size, which was associated with a decrease in cell size rather than total cell numbers. This suggests a role for *dS6K* in regulating cell growth in individuals that reach adulthood [59].

Similar to *Drosophila*, S6K1^−/−^ mice exhibit defects such as small size, hypoinsulinemia and glucose intolerance associated with decreased pancreatic beta cell size [60]. It has been observed that there is an upregulation of S6K2 expression in tissues from S6K1 knockout mice, which compensates for the decrease in rpS6 phosphorylation in these mice [61]. Conversely, rpS6 phosphorylation is abrogated in S6K2^−/−^ mice, suggesting that physiologically S6K2 is the principal kinase for rpS6. While S6K1/S6K2 double knockout mice exhibit perinatal lethality, S6K2^−/−^ mice survive to adulthood with no apparent phenotype [61].

What then is the physiological role of the highly conserved rpS6 phosphorylation? The mitogen-inducible phosphorylation of rpS6 occurs at five C-terminal serine residues [62] and is mediated by several distinct kinases [63]. In vivo knock-in mouse models that harbor a non-phosphorylatable mutant of rpS6 showed small size, hypoinsulinemia, decreased beta cell size and muscle weakness—phenotypes similar to those of S6K1 knockout mice [64,65]—which is counterintuitive since S6K2 seems to be the primary kinase mediating rpS6 phosphorylation [64]. Several explanations have been put forth to resolve this discrepancy. For example, given their distinct localization patterns, a pool of rpS6 that is accessible only by S6K1 may be required for mediating cell growth. Similarly, S6K1 but not S6K2 may be activated during a particular developmental stage when rpS6 phosphorylation is required [21]. Further studies in mice deficient for rpS6 phosphorylation revealed that this highly conserved phosphorylation event was dispensable for the translation of 5′ TOP mRNAs, an event long considered to depend on it [64].

Although originally identified as the kinase mediating rpS6 phosphorylation, several other cellular substrates of S6K, specifically S6K1, have since been reported that play important roles in gene transcription, protein translation, insulin resistance and cell survival. For example, S6K1 has been reported to phosphorylate and regulate components of the translation apparatus, such as eIF4B [66] and eEF2K [67], and regulators of protein synthesis, such as programmed cell death 4 (PDCD4), which inhibits the translation machinery [68]. It is believed to regulate cell survival via the phosphorylation and regulation of murine double minute 2 (MDM2) [69], a negative regulator of p53, and BAD [70], a cell death-promoting protein.

While glucose intolerance has been observed in S6K1^−/−^ mice, it has also been shown to promote the phosphorylation and feedback inhibition of insulin receptor substrate 1 (IRS1) [71,72,73,74]. Insulin- and amino acid-mediated activation of mTOR/S6K via the PI3K pathway leads to a negative feedback loop resulting in the phosphorylation and downregulation of IRS1 by S6K1 and eventually insulin resistance and type 2 diabetes. In addition to diabetes, mTOR/S6K1-mediated feedback inhibition of IRS1 and by extension the oncogenic PI3K/Akt pathway poses drawbacks for cancer therapy and limits the cytotoxic effects of rapamycin-based therapeutic approaches [75]. In addition to its role in the inhibition of IRS1, S6K1 has also been implicated in the phosphorylation and negative regulation of rictor, a component of the mTORC2 complex, which mediates Akt activation, although this conclusion remains controversial [76,77,78,79].

While several studies have documented the cellular functions and substrates of S6K1, little is known about those of S6K2. Given the lack of a phenotype in S6K2^−/−^ mice, the physiological role of S6K2 is poorly understood. Some reports suggest that it could play roles in cell proliferation and gene regulation via interaction with heterogeneous nuclear ribonucleoproteins (hnRNPs) [80], histone H3 [81] and ying-yang-1 (YY1) [82].

## 5. S6 Kinases and Breast Cancer

### 5.1. Gene Amplification of S6Ks in Breast Cancer

The first clue regarding the involvement of S6K1 in breast cancer came from the identification that S6K1 (encoded by *RPS6KB1*) is localized on the chromosomal region 17q23, which is amplified in 20% of primary breast cancers [83]. cDNA microarray analyses showed that S6K1 is amplified and overexpressed in MCF-7 breast cancer cells. Tissue microarray analysis of 668 primary breast tumors showed amplification of S6K1 in 8.8% of primary tumors [83]. Co-amplification of S6K1 and HER-2 located in chromosome 17q12 was associated with poor patient survival [83].

S6K2 encoded by the gene *RPS6KB2* was shown to be located on chromosome 11q13, which harbors several key mediators of breast cancer [84]. Perez-Tenorio et al., demonstrated that both *RPS6KB1* and *RPS6KB2* are often amplified in breast cancer tissues [84]. *RPS6KB1* amplification (≥4 copies) has been reported in 10.7% of breast cancers, and gene gains (≥3 copies) have been reported in 21.4% of breast cancers [84]. Furthermore, this has been associated with loco-regional recurrence [85]. While amplification of *RPS6KB2* is only associated with 4.3% of breast cancers, a large number of samples (21.3%) exhibit gains, suggesting that *RPS6KB2* gain rather than amplification is a major event in breast cancer [21]. A co-amplification of *RPS6KB2* and *4EBP1* has been reported, suggesting a synergy between these mTOR targets in breast cancer development and progression [86].

### 5.2. Expression and Localization of S6Ks in Breast Cancer

Immunohistochemical analysis demonstrated that both S6K1 and S6K2 are overexpressed in breast cancer, with S6K1 being primarily cytosolic and S6K2 predominantly nuclear in localization [87,88]. Furthermore, nuclear S6K2 correlated with staining of proliferation markers such as Ki-67 and proliferating cell nuclear antigen (PCNA), suggesting a role for nuclear S6K2 in breast cancer cell proliferation [87]. Additionally, nuclear accumulation of S6K2 was increased in cells at the periphery of the tumor, suggesting a unique role in breast cancer pathogenesis. However, Bostner et al., reported that high nuclear S6K1 was indicative of reduced benefits from tamoxifen treatment [89]. A recent study suggests that the subcellular distribution of S6K1 depends on the cell density and cell motility [90]. For example, at low cell density S6K1 was predominantly nuclear but it relocalized to the cytoplasm in confluent monolayers. During cell migration, S6K1 translocated to the nucleus and interacted with the transcription factor TBR2 (T-box brain protein 2). This study implicates nucleocytoplasmic shuttling of S6K1 to play an important role in the migration and invasion of breast cancer.

### 5.3. Function of S6Ks in Breast Cancer

#### 5.3.1. Involvement of S6Ks in Estrogen Receptor (ER)-Positive Breast Cancer

Estrogen receptor-α (ERα)-positive breast cancers account for over half of all breast cancers and hence constitute the major subtype [91]. The canonical or genomic ER signaling is characterized by the binding of estrogen and subsequent activation of ERα, which then translocates to the nucleus and regulates its target genes by either promoting or repressing their transcription [92]. Activation of ERα is associated with its phosphorylation by several different kinases including S6K1 [93,94,95]. Further studies showed that S6K1 and ERα constitute a positive feed-forward loop, where the phosphorylation of ERα by S6K1 promotes its activity, which in turn promotes transcription of *RPS6KB1* to mediate breast cancer cell proliferation [96,97]. The insulin-like growth factor (IGF) pathway plays a critical role in breast cancer. It was shown that knockdown/inhibition of S6K1 prevented IGF (insulin-like growth factor)-induced phosphorylation of ERα at Ser167 and transcription of ERα-regulated genes [98]. It has been reported that S6K1 mediates the phosphorylation of histone deacetylase 1 (HDAC1) by mitogens, recruitment of HDAC1 to the ERα promoter and increases in ERα transcription in breast cancer cells [99]. While the role and regulation of S6K1 in breast cancer have been addressed, little is known about the causes and consequences of S6K2 overexpression.

The availability of anti-estrogen therapies suggests a favorable prognosis for patients with ER-positive breast cancers. However, a substantial number of patients fail to respond to therapy [100], and the development of resistance further complicates treatment [91]. Studies in breast cancer tissues reported that *RPS6KB2* gains/amplifications correlate with ER-positivity [84]. Also, distant recurrence-free survival was substantially reduced in patients with ER-positive tumors associated with *RPS6KB2* gains/amplifications [84]. Furthermore, 11q13 amplifications have been intimately linked to ER-positive breast cancers [86,101,102,103,104] and constitute a high-risk subgroup of ER-positive cancers [101], suggesting that this region may play an important role in the response and resistance of breast cancer cells to death induced by anti-estrogen therapy. S6K2 but not S6K1 is frequently co-expressed with 4E-BP1, and high mRNA levels of S6K2 and/or 4E-BP1 mRNA were associated with poor prognosis and endocrine resistance in randomized Stockholm tamoxifen trials of four different cohorts of breast cancer patients [105]. Kim et al., proposed that phospho-S6K1 status is associated with poor prognosis and endocrine resistance of hormone receptor-positive breast cancers but not hormone receptor-negative breast cancers [106]. This is consistent with two other independent clinical trials demonstrating that S6K1 was associated with reduced response to tamoxifen [89]. While high levels of S6K1 correlated with markers of increased proliferation, HER2 status and cytoplasmic Akt, high levels of S6K2 correlated with low proliferation, ER status and nuclear Akt [89]. However, Ma et al., reported that although the levels of p-mTOR, p-4E-BP1 and p-S6K1 were significantly higher in breast tumor tissues compared to normal tissues, the status of phosphorylated mTOR, 4E-BP1 and S6K1 could not serve as prognostic factors for breast cancer [107]. The disease state (early versus late), node positivity, tumor size and treatment regime may influence the outcome of the biomarker studies.

Whole genome expression profiling of S6K1, S6K2 and 4E-BP1 in breast tumors also suggested distinct roles of S6K1 and S6K2 in breast cancer [26]. Knockdown of S6K2 but not S6K1 caused upregulation of genes associated with metabolism and regulation of cell cycle progression and checkpoints in ER-positive ZR751 cells. Knockdown of S6K1 caused upregulation of S6K2 and vice versa. Moreover, S6K2 knockdown caused an increase in raptor, whereas silencing of both S6K1 and S6K2 caused increases in mTOR and rictor, suggesting a compensatory cross-talk between the mTORC1 and mTORC2 complexes [26].

#### 5.3.2. Involvement of S6Ks in Triple-Negative Breast Cancer

S6K1 has also been associated with triple-negative breast cancer (TNBC). Estrogen-related receptor-α (ERRα), a member of the orphan nuclear receptor family, is closely related to ERα and plays an important role in cellular metabolism [108]. While ERRα has been associated with endocrine resistance, ERRα level could also predict tamoxifen sensitivity in TNBC [109]. TNBC cells express high levels of ERRα, and it was shown to negatively regulate S6K1 expression by directly binding to its promoter [110]. Knockdown/inhibition of ERRα enhanced S6K1 level and sensitized cells to rapamycin or S6K1 inhibitor to inhibit cell proliferation, migration, invasion and metastasis, and decreased the expression of the pro-survival proteins survivin and myeloid cell leukemia 1 (Mcl-1). PF-4708671, a pharmacological inhibitor of S6K1, inhibited cell migration in a highly metastatic variant of MDA-MB-231 cells [111]. In addition, miRNA-15/16 was shown to inhibit cell proliferation, induce apoptosis and inhibit epithelial to mesenchymal transition (EMT) in TNBC MDA-MB-231 cells by targeting *RPS6KB1* through binding to its 3′-UTR [112]. The miRNAs *miR-96*, *miR-557* and *miR-3182* were also shown to downregulate S6K1 by targeting the 3′-UTR of S6K1 mRNA [113].

Although the involvement of S6K2 in breast cancer metastasis has not been studied yet, S6K2 was shown to be a direct target of *miR-193a-3p*, which suppresses lung metastasis; downregulation of S6K2 by *miR-193a-3p* was shown to be a potential mechanism by which *miR-193a-3p* inhibited migration, invasion and EMT in non-small cell lung cancer [55]. On the other hand, upregulation of S6K2 was implicated in mediating the effects of *miR-1273g-3p* on cell proliferation, migration and invasion of colorectal cancer cells [114]. Based on analysis of the TCGA dataset, S6K2 but not S6K1 was overexpressed in both ER-positive and TNBC breast tumor tissues [115]. Moreover, overexpression of S6K2 in TNBC cells attenuated cell death by apoptosis [115].

#### 5.3.3. Regulation of Apoptosis by S6Ks

Both S6K1 and S6K2 have been implicated in regulating cell death by apoptosis. While S6K1 inhibitor PF-4708671 alone did not influence the levels of anti-apoptotic proteins, it decreased Mcl-1 and survivin levels when MCF-7 cells were deprived of glucose [116] or treated in combination with tamoxifen [117]. PF-4708671 in combination with Bcl-2 inhibitor ABT263 also decreased survivin levels but increased Mcl-1 levels in BT474 cells [118]. In this study, the effect of S6K1 knockdown on the levels of anti-apoptotic proteins was not examined. Although PF-4708671 is specific towards S6K1 at low concentrations, it could inhibit MSK1, AMPK, RSK as well as S6K2 at high concentrations [119]. Moreover, PF-4708671 could phosphorylate S6K1 at Thr229 and Thr389, resulting in its activation [119].

We have shown that knockdown of S6K2 alone but not S6K1 sensitized cells to apoptotic stimuli by altering the ratio of pro- and anti-apoptotic proteins [27,120]. We made an important observation that S6K2 cooperates with Akt in mediating breast cancer cell survival [27]. While knockdown of S6K1 caused activation of Akt and inhibited cell death by apoptosis, knockdown of S6K2 decreased Akt activation and increased cell death by TNF and TRAIL by enhancing the cleavage of the pro-apoptotic Bcl-2 family protein Bid [27]. S6K2 could also promote breast cancer cell survival via an Akt-independent but JNK (c-Jun N-terminal kinase)-dependent pathway [120]. Knockdown of S6K2 decreased both Bcl-xl and Mcl-1 in T47D cells. While S6K2 appears to regulate Bcl-xl via the tumor suppressor PDCD4 [121], it positively regulated Mcl-1 via a JNK-dependent but GSK3-independent pathway [120].

#### 5.3.4. The Involvement of Long and Short S6K Isoforms in Breast Cancer

There are controversies regarding which isoform of S6K1 is involved in breast cancer. It has been reported that the short kinase-inactive splice variants of S6K1 contribute to breast cancer, whereas the long p85/p70 S6K1 form causes tumor suppression [122]. The short isoforms were overexpressed in breast cancer cells and tissues and exhibited their oncogenic properties partly by causing activation of mTORC1 and increases in 4E-BP1 phosphorylation, cap-dependent translation and Mcl-1 levels. A recent study showed that the long p85 S6K1 but not p70 S6K1 or p56 S6K2 is secreted from cancer cells and can enter surrounding cells to promote breast cancer cell growth, migration and invasion [123]. Moreover, injection of recombinant p85 S6K1 in mice enhanced tumorigenesis and lung metastasis in an MDA-MB-231 tumor xenograft [123].

## 6. Conclusions

Given the role of mTOR in promoting protein translation, cell growth and proliferation, it is an attractive target for cancer therapy [124]. However, S6K1 and S6K2, the downstream targets of mTORC1, carry out distinct functions. While S6K1 has a more prominent role in regulating cell proliferation, invasion and metastasis, S6K2 appears to have a greater impact on cell death regulation. Rapamycin and its analogs that inhibit mTORC1 are being evaluated for their clinical potential in several cancers, including breast cancer [125,126]. Furthermore, due to the association between *RPS6KB1* amplifications and cancer, there has been considerable interest in the development of inhibitors of S6K1, such as LYS6K2 [127] and PF-4708671 [119]. However, Ly2584702 tosylate, an inhibitor of S6K1 that is not an analog of rapamycin, was ineffective as a single agent [128] and was not well tolerated when administered in combination with everolimus and erlotinib [129] in phase I clinical trials. The therapeutic efficacy of mTORC1 and S6K1 inhibitors is also thwarted due to the existence of a negative feedback loop between PI3K/Akt and mTOR/S6K1 signaling [75]. Persistent inhibition of S6K1 leads to the activation of PI3K/Akt, allowing survival of cancer cells [130,131,132,133]. Inhibition of mTORC1 can also cause a compensatory activation of the MAPK pathway [134]. Phosphorylation of Grb10, a substrate for mTORC1, leads to feedback inhibition of both the PI3K/Akt and MAPK pathways [135,136]. These observations dampen the enthusiasm for mTORC1- and S6K1-inhibitor-based approaches in cancer therapy. Currently, combinatorial approaches using dual-specificity inhibitors of PI3K/Akt and mTOR are being evaluated [137]. Furthermore, the observation that S6K1^−/−^ mice are characterized by small size and exhibit hypoinsulinemia suggests that targeting S6K1 for cancer therapy may be associated with significant side effects [60]. The normal development and the lack of an apparent phenotype in S6K2^−/−^ mice suggests that it is a potential target in the treatment of endometrial [138], gastric [139] and breast cancers [84,87,88], which have been shown to have *RPS6KB2* amplification or elevated S6K2 expression. Moreover, knockdown of S6K2 resulted in inhibition rather than activation of Akt in breast cancer cells [27]. Thus, there should be an emphasis on the development of S6K2-specific inhibitors. Even though the kinase domains of S6K1 and S6K2 are similar, the unique amino acids at the ATP binding pocket of S6K1 (Tyr) and S6K2 (Cys) may allow development of S6K2-specific inhibitors [26]. Further mechanistic studies dissecting the function of S6K1 and S6K2 at various stages and types of breast cancer are needed in order to properly exploit these two homologs for breast cancer therapy.

## Figures and Tables

**Table 1:** Genes and isoforms of the 40S ribosomal S6 kinases (S6Ks).

Gene	Chromosome	Isoforms	Size
*RPS6KB1*	17	S6KαI	p85
		S6KαII *	p70
*RPS6KB2*	11	S6KβI	p56
		S6KβII *	p54

* Predominant isoform.

## Abbreviations

4E-BP1	Eukaryotic translation initiation factor 4E (eIF4E)-binding protein 1
EMT	Epithelial–mesenchymal transition
ERα	Estrogen receptor-α
ERRα	Estrogen-related receptor-α
HDAC	Histone deacetylase
hnRNP	Heterogeneous nuclear ribonucleoprotein
IGF	Insulin-like growth factor
IRS1	Insulin receptor substrate 1
MAPK	Mitogen-activated protein kinase
Mcl-1	Myeloid cell leukemia 1
MDM2	Murine double minute 2
MEK	Mitogen-activated protein kinase kinase
mTOR	Mechanistic target of rapamycin
NLS	Nuclear localization signal
PDCD4	Programmed cell death 4
PDK1	3-Phosphoinositide-dependent protein kinase 1
PDZ	Post synaptic density protein; PSD95, Drosophila disc large tumor suppressor; Dlg1, and Zonula occludens-1 protein; zo-1
PHLPP	Pleckstrin homology domain leucine-rich repeat protein phosphatase
PI3K	Phosphatidylinositol 3-kinase
PKC	Protein kinase C
S6K	40S ribosomal S6 kinase
TNBC	Triple-negative breast cancer
TOS	Tor signaling motif
TSC	Tuberous sclerosis complex
YY1	Ying-yang-1
